# Human α-Defensin-5 Efficiently Neutralizes *Clostridioides difficile* Toxins TcdA, TcdB, and CDT

**DOI:** 10.3389/fphar.2020.01204

**Published:** 2020-08-12

**Authors:** Michael Korbmacher, Stephan Fischer, Marc Landenberger, Panagiotis Papatheodorou, Klaus Aktories, Holger Barth

**Affiliations:** ^1^ Institute of Pharmacology and Toxicology, University of Ulm Medical Center, Ulm, Germany; ^2^ Institute of Experimental and Clinical Pharmacology and Toxicology, University of Freiburg, Freiburg, Germany

**Keywords:** *C. difficile* infection, large clostridial glucosylating toxins, binary actin ADP-ribosylating toxin, toxin inhibitor, AB-type protein toxins

## Abstract

Infections with the pathogenic bacterium *Clostridioides (C.) difficile* are coming more into focus, in particular in hospitalized patients after antibiotic treatment. *C. difficile* produces the exotoxins TcdA and TcdB. Since some years, hypervirulent strains are described, which produce in addition the binary actin ADP-ribosylating toxin CDT. These strains are associated with more severe clinical presentations and increased morbidity and frequency. Once in the cytosol of their target cells, the catalytic domains of TcdA and TcdB glucosylate and thereby inactivate small Rho-GTPases whereas the enzyme subunit of CDT ADP-ribosylates G-actin. Thus, enzymatic activity of the toxins leads to destruction of the cytoskeleton and breakdown of the epidermal gut barrier integrity. This causes clinical symptoms ranging from mild diarrhea to life-threatening pseudomembranous colitis. Therefore, pharmacological inhibition of the secreted toxins is of peculiar medical interest. Here, we investigated the neutralizing effect of the human antimicrobial peptide α-defensin-5 toward TcdA, TcdB, and CDT in human cells. The toxin-neutralizing effects of α-defensin-5 toward TcdA, TcdB, and CDT as well as their medically relevant combination were demonstrated by analyzing toxins-induced changes in cell morphology, intracellular substrate modification, and decrease of trans-epithelial electrical resistance. For TcdA, the underlying mode of inhibition is most likely based on the formation of inactive toxin-defensin-aggregates whereas for CDT, the binding- and transport-component might be influenced. The application of α-defensin-5 delayed intoxication of cells in a time- and concentration-dependent manner. Due to its effect on the toxins, α-defensin-5 should be considered as a candidate to treat severe *C. difficile*–associated diseases.

## Introduction

Bacterial AB-type protein toxins belong to the most toxic substances in nature and are able to cause a broad variety of severe diseases in humans and animals. The extraordinary potency of bacterial toxins is based on their inimitable structures harboring enzyme activities and their highly sophisticated uptake mechanisms (Schiavo and van der Goot, 2001; Uptake and Trafficking of Protein Toxins | Holger Barth | Springer). The toxins serve as important virulence factors, which are directly linked to the clinical symptoms of human diseases as for example diphtheria, anthrax or other severe enteric complications such as medically relevant *Clostridioides* (*C.*, formerly *Clostridium*) *difficile*–associated diseases, in particular diarrhea (CDAD). Especially *C. difficile* infection (CDI) remains a remarkable challenge for affected patients and global health care systems. The characteristic symptoms for CDI range from mild and watery diarrhea up to severe forms of pseudomembranous or fulminant colitis which may ultimately end in multi-organ failure (Spencer, 1998; Goudarzi et al., 2014). In addition to the patients suffering, CDIs also remain a high economic burden. In England, the costs for CDIs have been estimated at €5000 - €15000 per case (Kuijper et al., 2006) accompanied with an increased length of stay in hospital (van Kleef et al., 2014; Wilcox et al., 2017).

The gram-positive, spore-forming anaerobic bacterium *C. difficile* is an important nosocomial gastrointestinal human gut pathogen. The incidence and severity of CDIs have dramatically increased over the last decade. This is mainly attributed to the emergence of new hypervirulent strains. First and foremost, the epidemically occurring *C. difficile* PCR ribotype O27 strain gained more and more attention. This strain is characterized by its comprehensive occurrence in Canada, the USA and continental Europe (Pépin et al., 2004; Pépin et al., 2005; Kuijper et al., 2008) and due to its higher morbidity and mortality mainly caused by the presence of the three proteinaceous AB-type toxins TcdA, TcdB, and CDT (Kuehne et al., 2014). In fact, not the bacterium itself is responsible for the development of clinical disease presentations but rather the produced and secreted AB-type protein toxins. They are responsible for epithelial breakdown of the gut barrier integrity resulting in severe enterotoxicity (Carter et al., 2012; Carter et al., 2015). The main causative determinants of *C. difficile* are the two large single-chain AB-type toxins TcdA and TcdB (Aktories, 2011). They alone are sufficient to develop the full disease pattern (Kuehne et al., 2010; Kuehne et al., 2014). TcdA and TcdB exhibit a high sequence homology and share the same multidomain architecture (von Eichel-Streiber et al., 1992; Jank and Aktories, 2008). However, hypervirulent *C. difficile* strains are able to produce in addition to TcdA and TcdB a third toxin, the binary toxin CDT (*C. difficile* transferase). CDT is a bipartite toxin and is comprised of the enzymatic active component CDTa and the binding- and translocation-component CDTb. CDTb is able to form heptamers to which one single CDTa-molecule can bind (Sheedlo et al., 2020). Although CDT differs in structure and function, all three toxins share some important similarities (Papatheodorou et al., 2018). They are secreted from the bacteria and enter their human target cells *via* receptor-mediated endocytosis. In acidified endosomes, their conformation changes and their catalytic domains are released into the host cell cytosol where they modify their specific intracellular target proteins. TcdA and TcdB glucosylate and thereby inactivate small GTPases of the Rho and Ras families of monomeric GTPases (Just et al., 1995a; Just et al., 1995b; Just et al., 1996) that cause colonic tissue damage by distinct mechanisms (Chumbler et al., 2016), whereas CDT acts as an ADP-ribosyltransferase modifying monomeric G-actin (Perelle et al., 1997; Gülke et al., 2001). Intracellular substrate modification leads to depolymerization of the actin cytoskeleton, and thus to cell rounding and breakdown of the intestinal gut barrier integrity. Therapy of CDI is challenging because an effective antibiotic treatment is mostly limited to broad-spectrum antibiotics such as metronidazole, vancomycin, or the newer anti-CDI drug fidaxomicin (Debast et al., 2014), as first-line treatment (Louie et al., 2011; Tart, 2013). Indeed, application of antibiotics might eventually result in further disturbance of the gut microbiota increasing the risk of recurrent CDIs.

Therefore, novel therapeutic approaches to treat CDI preferentially based on the inactivation of the produced and secreted toxins are urgently needed. In this context, antimicrobial peptides (AMPs) and in particular human defensins play an elevated role. Defensins are small and cationic peptides linked *via* three intra-molecular disulfide bridges (Ganz and Lehrer, 1994; Kagan et al., 1994). In addition to mere microbicidal activity, inactivation and neutralization of several bacterial toxins were reported (Kim et al., 2005; Kim et al., 2006; Giesemann et al., 2008; Lehrer et al., 2009). Especially for α-defensins, an inhibitory potency against several bacterial toxins was reported earlier (Kim et al., 2005; Kim et al., 2006; Giesemann et al., 2008; Fischer et al., 2018; Fischer et al., 2020). Based on these findings, we investigated the effect of the human antimicrobial peptide α-defensin-5 as inhibitor of the *C. difficile* toxins TcdA, TcdB, and CDT and in particular as inhibitor of the medically most relevant combination of all three toxins.

## Materials and Methods

### Protein Expression, Purification, and Used Inhibitor

The recombinant protein toxins used in this work were expressed and purified as described in earlier publications (Schwan et al., 2009; Papatheodorou et al., 2010; Schwan et al., 2011). Native TcdA was purified as described (Giesemann et al., 2008). α-Defensin-5 was purchased from PeptaNova (Sandhausen, Germany) and dissolved as described by the manufacturer.

### Cell Culture and Cytotoxicity Experiments

Vero and Caco-2 cells were cultivated in culture dishes at 37°C and 5% CO_2_. For cultivation, cells were maintained in their respective media (Vero cells: Minimum Essential Media (MEM) containing 10% fetal calf serum (FCS), 1 mM sodium pyruvate, 2 mM l-glutamine, 0.1 mM non-essential amino acids (NEAA), 10 g/L penicillin/streptomycin; Caco-2 cells: Dulbecco’s Modified Eagle’s Medium (DMEM) containing 10% FCS, 1 mM sodium pyruvate, 0.1 mM NEAA, 10 g/L penicillin/streptomycin) and split three times a week at a confluency of 80% to 100%. For cytotoxicity experiments, cells were seeded into different well plates ranging from 96- to 8-well cell culture plates and grown for at least 1 day. After reaching the requested density, the cells were treated with the respective toxins in serum-free medium. After defined time points, pictures were taken using an Axiovert 40CFl microscope from Zeiss connected to a ProgRes C10 CCD camera from Jenoptik to monitor the intoxication process. Cell pictures were processed using the ImageJ software (Schneider et al., 2012; Rueden et al., 2017) in combination with the plug in cell_counter.jar.

### Investigation of the Glucosylation Status of Rac1 in Cells After Treatment With TcdA and/or TcdB

After reaching confluency, cells were treated with either TcdA (10 pM), TcdB (10 pM) or the combination of both toxins (each 10 pM) in the presence or absence of α-defensin-5 in concentrations ranging from 1 µM to 6 µM. For control, cells were left untreated. After defined time points, the cells were thoroughly washed, lysed, and transferred to SDS-PAGE followed by Western blot. Native and thereby non-glucosylated Rac1 was detected by using an anti-Rac1-antibody (BD, Bioscience, 610650, 1:1000) combined with the respective secondary antibody (chicken-anti-mouse IgG-HRP, Santa Cruz Biotechnology, 1:2500). Comparable protein loading of the samples was confirmed *via* immunodetection of either Hsp90 (Santa Cruz Biotechnology, 1:500) or GAPDH (Santa Cruz Biotechnology, 1:1000).

### Sequential ADP-Ribosylation of Actin in Lysates of CDT-Treated Cells

After reaching confluency, cells were treated with CDT (CDTa/CDTb: 1 nM/1.3 nM) in the presence or absence of α-defensin-5 (1, 3, or 6 µM). For control, cells were left untreated. After 4.5 h, cells were washed and scraped off in 50 µl of ADP-ribosylation-buffer containing 1 mM DTT, 5 mM MgCl_2_ and 1 mM EDTA, 20 mM Tris-HCl pH 7.5 plus cOmplete™ protease inhibitor cocktail (Roche, Germany). After lysis, 20 µl of the cell lysate was incubated with 10 µM biotinylated NAD^+^ (Trevigen, USA) in the presence of 50 ng freshly added CDTa for 30 min at 37°C. After adding SDS sample buffer and inactivation for 10 min at 95°C, samples were analyzed by Western blotting. Biotin-labeled, i.e., ADP-ribosylated actin was detected with a peroxidase-coupled streptavidin (Sigma-Aldrich, USA, 1:2500). To ensure comparable protein loading, GAPDH was detected as described above.

### *In Vitro* Glucosylation of Rac1 by TcdA and *In Vitro* ADP-Ribosylation of Actin by CDTa

For investigation of the *in vitro* glucosylation of Rac1, 40 µg of Caco-2 whole cell lysate was incubated with 300 ng TcdA for 1 h at 37°C in the presence of increasing concentration of α-defensin-5 (6, 12, or 24 µM). For control, cell lysate was either left untreated or incubated with TcdA alone. After heat inactivation, samples were transferred to SDS-PAGE followed by Rac1 immunoblotting. For investigation of the *in vitro* ADP-ribosylation of actin, 40 µg of whole Caco-2 lysate was incubated with 1 ng CDTa for 30 min at 37°C with 10 µM biotinylated NAD^+^ and with increasing concentrations of α-defensin-5 (1 µM, 3 µM, and 6 µM). For control, cell lysate was supplemented with 10 µM biotinylated NAD^+^, with CDTa of was left untreated. Biotin-labeled, i.e. ADP-ribosylated actin was detected as mentioned above.

### Fluorescence Microscopy

**Figure 1:**
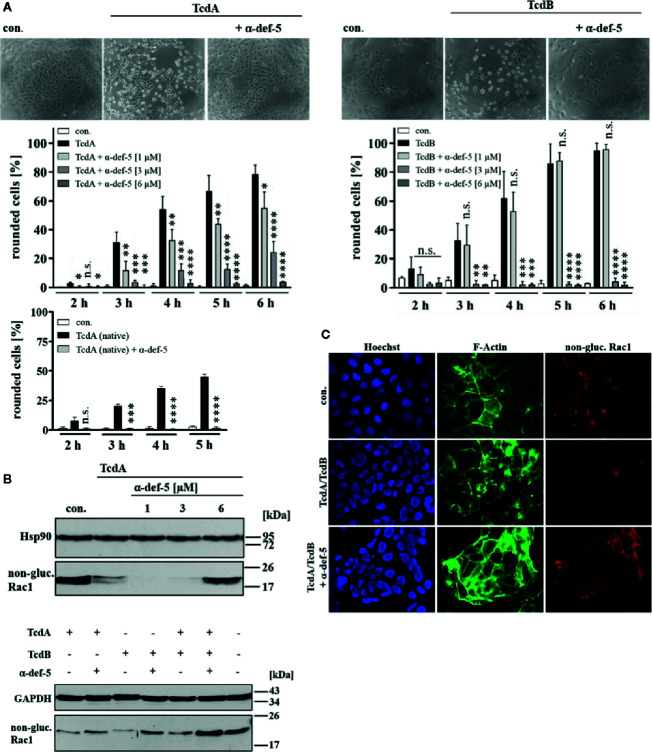
α-Defensin-5 decreases the cytotoxic effects of TcdA, TcdB and of the combination of both toxins. **(A)** Vero cells were either treated with TcdA (10 pM, left panel) or TcdB (10 pM, right panel) in the presence or absence of increasing concentrations of α-defensin-5 (1, 3, 6 µM), and the percentage or rounded cells was determined. For comparison, Vero cells were treated with native TcdA (10 pM, left lower panel) in the presence or absence of α-defensin-5 (6 µM). Values are given as mean ± SD (n = 3). Significance was determined using the one-way ANOVA test (n.s. = not significant, *p < 0.05, **p < 0.01, ***p < 0.001, ****p < 0.0001). **(B)** Vero cells (upper panel) were treated with TcdA (10 pM) and α-defensin-5 with increasing concentrations (1, 3, 6 µM) for 3.5 h. Caco-2 cells (lower panel) were treated with either TcdA (10 pM), TcdB (10 pM) or the combination of both toxins (each 10 pM) with or without α-defensin-5 (6 µM) for 8 h. Afterward, cells were lysed and subjected to Western blot analysis. Non-glucosylated Rac1 was detected using a specific antibody. Hsp90 or GAPDH were used as controls for equal protein loading. **(C)** Caco-2 cells were treated with the combination of TcdA (10 pM) plus TcdB (10 pM) and with/without α-defensin-5 (6 µM) for 8.5 h. For control, cells were left untreated. After incubation, cells were fixed and permeabilized. Non-glucosylated Rac1 was detected using a specific anti-Rac1-antibody, F-actin was stained with phalloidin-FITC, nuclei were stained with Hoechst33342.

**Figure 2:**
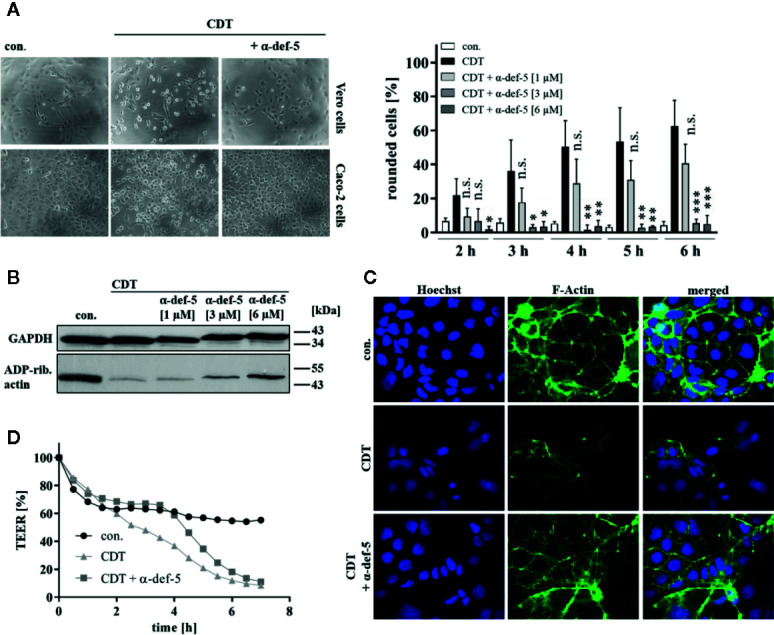
α-Defensin-5 protects cells from intoxication with the binary toxin CDT. **(A)** Vero cells (upper left panel) were treated with CDT (1 nM/1.3 nM) and Caco-2 cells (lower left panel) were treated with CDT (4.1 nM/5.3 nM) in the presence or absence of α-defensin-5 with varying concentrations. Representative images for Vero cells (6 h) and Caco-2 cells (4.5 h) plus/minus α-defensin-5 (6 µM) are depicted. For Vero cells, the amount of rounded cells over time was determined (right panel). Values are given as mean ± SD (n = 3). Significance was determined using the one-way ANOVA test (n.s. = not significant, *p < 0.05, **p < 0.01, ***p < 0.001). **(B)** Caco-2 cells were treated for 4.5 h with CDT (4.1 nM/5.3 nM) with and without α-defensin-5 (6 µM). Afterward, cells were washed, lysed and subjected with 10 µM biotinylated NAD^+^ and 50 ng fresh CDTa. Biotin-labeled, e.g. ADP-ribosylated actin was detected by immunoblotting using the ECL system. GAPDH was stained for comparable protein loading. **(C)** Caco-2 cells were treated with CDT (2 nM/2.7 nM) with or without α-defensin-5 (6 µM) for 5 h. For control, cells were left untreated. Then, cells were fixed and permeabilized. Phalloidin-FITC was used to stain F-actin, Hoechst33342 was used to stain nuclei. **(D)** Transepithelial electrical resistance was investigated using Caco-2 cells with CDT (1.6 nM/2 nM) with or without α-defensin-5 (6 µM).

**Figure 3:**
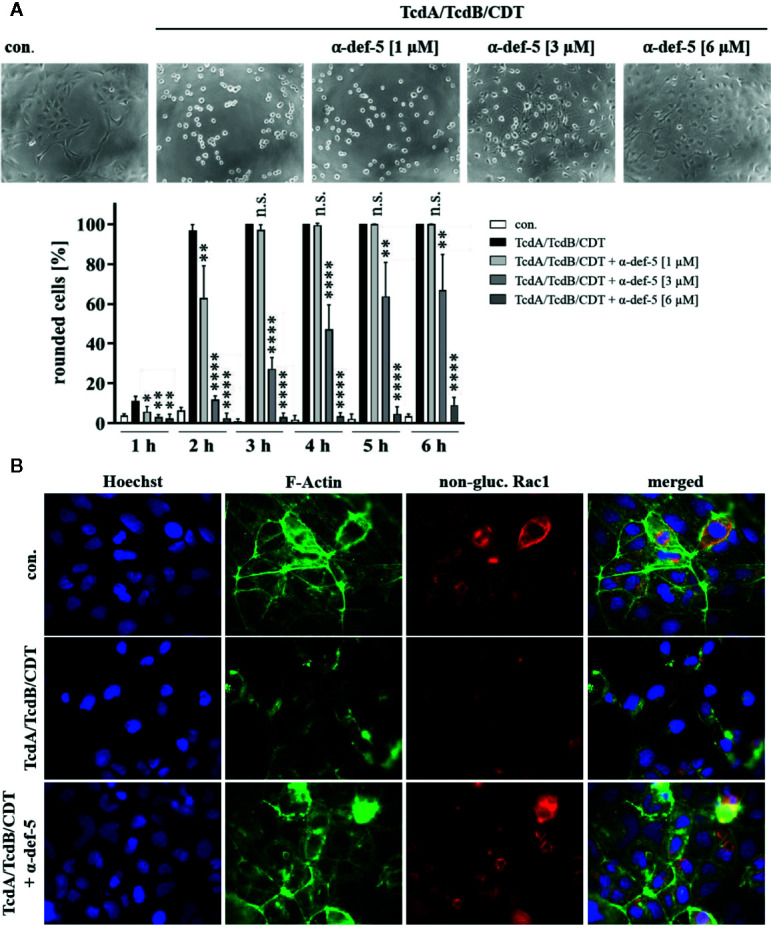
α-Defensin-5 protects cells in a time- and concentration-dependent manner from intoxication with the combination of TcdA, TcdB, and CDT. **(A)** Vero cells were treated with the combination of all three *C. difficile* toxins (TcdA: 10 pM, TcdB: 10 pM, CDT: 1 nM/1. 3 nM) and increasing concentrations of α-defensin-5 (1, 3, 6 µM). Representative images after 6 h are shown (upper panel). The amount of rounded cells was determined over time (lower panel). Values are given as mean ± SD (n=3). Significance was determined using the one-way ANOVA test (n.s. = not significant, *p < 0.05, **p < 0.01, ****p < 0.0001). **(B)** Caco-2 cells were treated with the combination of TcdA (10 pM), TcdB (10 pM), and CDT (2 nM/2.7 nM) in the presence or absence of α-defensin-5 (6 µM). After 5.5 h, cells were fixed, permeabilized and non-glucosylated Rac1 was stained using a specific antibody. Phalloidin-FITC was used to stain F-actin, Hoechst33342 was used to stain nuclei.

Caco-2 cells were seeded in 8-well µ-slide chambers from ibidi (Gräfelfing, Germany) and incubated for 2 days at 37°C until reaching confluency. Afterward, cells were treated with either the combination of TcdA/TcdB (each 10 pM), CDT (2 nM/2.7 nM), or the combination of all three toxins together (TcdA: 10 pM, TcdB: 10 pM, CDT: 2 nM/2.7 nM) in the presence or absence of α-defensin-5 (6 µM). After indicated time intervals of incubation, cells were washed carefully two times with PBS and fixed using 4% paraformaldehyde (PFA) for 20 min at room temperature (RT). After permeabilization with Triton-X 100 (0.4% in PBS) for 5 min and treatment with 100 mM glycine for 2 min at RT, cells were incubated with 5% skim milk powder for 30 min at 37°C. Non-glucosylated Rac1 was stained with a mouse anti-Rac1 antibody (BD Bioscience, 1:100) in combination with a fluorescent labeled secondary antibody (goat-anti-mouse-568, Invitrogen, USA, 1:750). Actin was stained using phalloidin-FITC (Sigma Aldrich, 1:100) and nuclei were stained *via* Hoechst33342 (1:10000) at 37°C for 5 min. Images were taken using iMic digital microscope (FEI, Munich, Germany) and processed using ImageJ software (following wavelength settings were used: **Figure 1C**: channel 1 (Hoechst), 500/1300; channel 2 (F-actin), 600/1200; channel 3 (Rac1), 650/950. **Figure 2C**: channel 1 (Hoechst), 500/1200; channel 2 (F-actin), 520/600; channel 3 (Rac1), 1000/1000. **Figure 3B**: channel 1 (Hoechst), 500/1300; channel 2 (F-actin), 515/600; channel 3 (Rac1), 515/600).

### TEER Measurements

Transepithelial electrical resistance (TEER) measurements were used to analyze the integrity of a confluent Caco-2 cell monolayer. Here, 1.2 × 10^5^ Caco-2 cells were seeded in a 24-well hanging cell culture insert (catalogue number MCHT24H48) from Merck Millipore and incubated for 3 days at 37°C until TEER values between 2000 and 3000 Ω/cm^2^ were reached. CDT (1.6 nM/2 nM) was added apically in complete growth medium in the presence or absence of α-defensin-5 (6 µM). TEER was measured using the EVOMX apparatus provided with the STX2 electrode (both WPI, USA). Blank resistance (filters only filled with culture medium) was subtracted from raw TEER values and resulting values were multiplied by the effective surface area of the membrane in the filter (here 0.3 cm^2^). Additionally, the exploited values were normalized to time point zero (t0 = 100%).

### Precipitation Studies With TcdA

After centrifugation at 10000 rpm for 20 min at 4°C, 1 µg of TcdA was incubated for 15 min at 37°C in 30 µl serum-free medium in the presence or absence of α-defensin-5 (6 µM). After an additional centrifugation step at 14000 rpm for 20 min at 4°C, samples were divided into a supernatant and a pellet fraction. The pellet fraction was resuspended in 30 µl MEM, and all fractions were incubated with SDS sample buffer at 95°C for 10 min. Afterward, the samples were transferred to and analyzed by SDS-PAGE.

### Calcium (Ca^2+^) Imaging

Caco-2 cells were seeded in an 8-well µ-slide plate from ibidi (Gräfelfing, Germany) with a density of 1.5 × 10^5^ cells per well for 2 days. Then, the cells were loaded with 3 µM of Fura-2 AM for 45 min at 37°C and afterward treated with bath solution (containing 140 mM NaCl, 5 mM KCl, 2 mM CaCl_2_, 1 mM MgCl_2_, 5 mM Glucose, 10 mM HEPES, pH 7.4). After three washing steps with bath solution, baseline was measured for 2 min, and cells were then treated with CDTb (13 nM) with or without α-defensin-5 (6 µM). Calcium flow was recorded using an iMic digital microscope (FEI, Munich, USA). Resulting ratio images were created with excitation light pulses at 340 and 380 nm followed by subsequent ratio calculations (340/380).

## Results

### The Human Peptide α-Defensin-5 Decreased the Cytotoxic Activities of TcdA and TcdB

First, the cytopathic effects of TcdA and TcdB under the influence of α-defensin-5 were examined in detail using the mammalian epithelial Vero cell line. After intoxication, Vero cells rapidly display a characteristic change in morphology, i.e. cell rounding, which is a traditional and well-established specific, robust, and sensitive endpoint to monitor the inhibition of bacterial protein toxins. The TcdA- and TcdB-induced changes in cell morphology (cell rounding) in the presence and absence of increasing concentrations of α-defensin-5 were quantified by cell counting. Representative images which clearly show a specific inhibition of TcdA and TcdB by α-defensin-5 are displayed (**Figure 1A**). Since the effect was surprising for TcdA, native toxin A was also tested in this case, which was also inhibited by α-defensin-5 (**Figure 1A**, left lower panel). All subsequent experiments were then performed with recombinant TcdA. In order to further strengthen the inhibitory potential of α-defensin-5 especially on TcdA, the status of Rac1 glucosylation in intoxicated cells was examined. Here, an antibody was used, that is only able to detect non-modified Rac1 from untreated cells. After intoxication and thereby glucosylation, Rac1 is no longer detected by this specific antibody (Genth et al., 2006: Egerer et al., 2007; Fischer et al., 2020). First, TcdA was investigated with increasing amounts of α-defensin-5 and a clear inhibition could be observed with the highest amount (6 µM) of the inhibitor (**Figure 1B**, upper panel). But also for TcdB and more importantly for the medically relevant combination of both toxins, a clear inhibition in the presence of α-defensin-5 could be detected (**Figure 1B**, lower panel). The results for the combination of both toxins were further confirmed *via* fluorescence microscopy (**Figure 1C**).

### Human α-Defensin-5 Decreased the Cytotoxic Activity of CDT

Next, the effect of α-defensin-5 toward the binary toxin CDT was examined. First, Vero as well as human colonic Caco-2 cells were treated with CDT in the presence and absence of α-defensin-5 and a clear time- and concentration-dependent inhibition became obvious (**Figure 2A**). Especially for the higher concentrations of the peptide, the inhibition of CDT could be clearly confirmed *via* investigating the effect on actin modification in CDT-treated Caco-2 cells (**Figure 2B**). After visualization of the actin cytoskeleton *via* fluorescence microscopy, it became evident, that CDT caused a dramatic disorganization of actin which is characterized by a lower actin signal in total. In the presence of α-defensin-5, the cytoskeleton was almost completely protected from CDT-catalyzed degradation (**Figure 2C**). Last, the integrity of the epithelial barrier function of confluently grown Caco-2 cells was analyzed. CDT-treatment clearly reduced TEER in the Caco-2 monolayer whereas this effect was delayed in the presence of α-defensin-5 (**Figure 2D**).

### Treatment With α-Defensin-5 Protected Cells From the Combination of TcdA, TcdB, and CDT

Now, the medically relevant combination of all three *C. difficile* toxins together was examined. Especially in antibiotic-resistant hypervirulent *C. difficile* strains, the presence of all three toxins leads to a significant worsened outcome for infected patients. In the cell culture experiments the combination of all three toxins led to a very rapid rounding of Vero cells. The characteristic rounding was significantly delayed in the presence of α-defensin-5 and also for the combination, a clear time- and concentration-dependent inhibition became evident (**Figure 3A**). To confirm the results obtained so far and to directly visualize effects of the combination of TcdA, TcdB, and CDT in intact Caco-2 cells, fluorescence microscopy was performed. Unimpaired Caco-2 cells are characterized by a clear ring of cortical actin and homogeneously distributed Rac1. After treatment with the toxins, these characteristics were dramatically altered. In the presence of α-defensin-5, actin as well as Rac1 was almost entirely protected from toxin-induced modifications (**Figure 3B**).

### Incubation of α-Defensin-5 With TcdA Resulted in Precipitation and With CDTb in Reduced Cytotoxicity and Pore Formation

**Figure 4:**
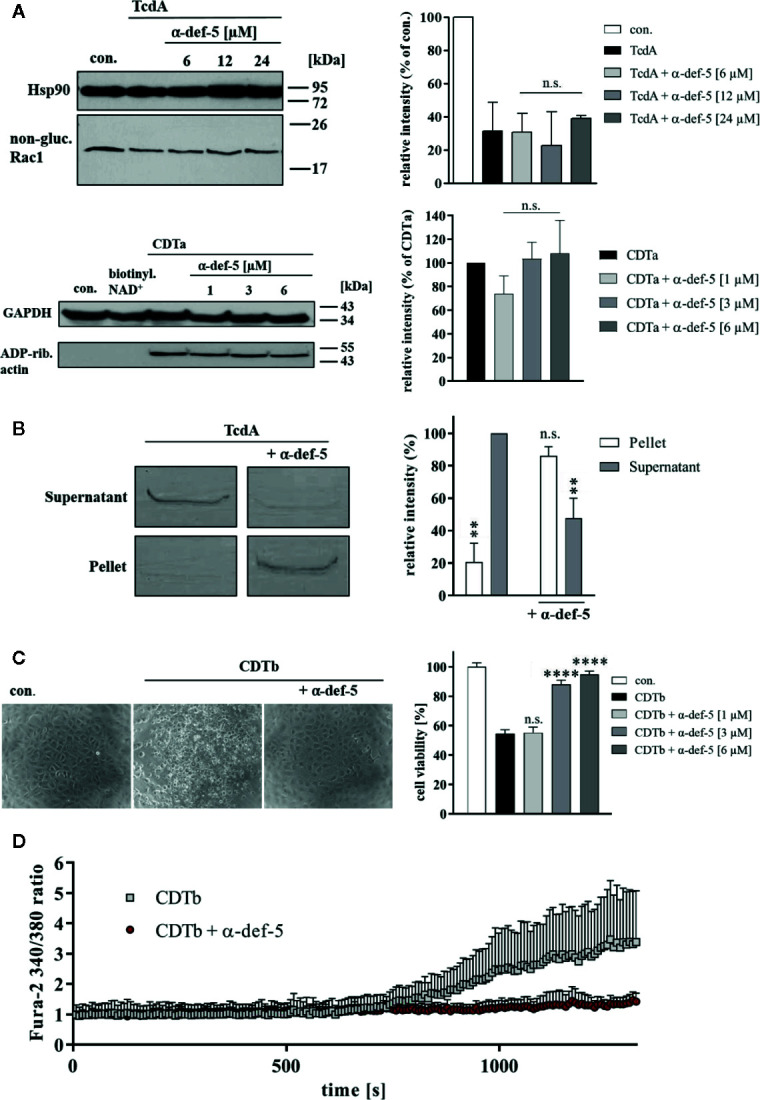
α-Defensin-5 has no influence on the enzymatic activity of TcdA and CDTa but leads to precipitation of TcdA and inhibition of the cytotoxic pore-forming activity of CDTb. **(A)** Caco-2 lysate (40 µg) was incubated with TcdA (300 ng) and varying concentrations of α-defensin-5 (6, 12, 24 µM) for 1 h (left panel) at 37°C. Caco-2 lysate (40 µg) was incubated with CDTa (1 ng), 10 µM biotinylated NAD^+^ and varying concentrations of α-defensin-5 (1, 3, 6 µM) for 30 min at 37°C. Next, samples were subjected to SDS-PAGE and Western Blotting. Non-glucosylated Rac1 was detected with a specific antibody, biotin-labeled, e.g., ADP-ribosylated actin was detected using streptavidin-peroxidase. Hsp90 and GAPDH were used to confirm equal protein loading. Values are given as mean ± SD (n = 2). Significance was determined using the one-way ANOVA test (n.s. = not significant). **(B)** TcdA (1 µg) was incubated with and without α-defensin-5 (6 µM) for 15 min at 37°C in serum-free medium. Afterward, samples were centrifuged and separated fractions were subjected to SDS-PAGE (left panel). Densitometric analyses from individual experiments are shown as bar graph (right panel). Values are given as mean ± SD (n = 2). Significance was determined using the one-way ANOVA test (n.s. = not significant, **p < 0.01). **(C)** Vero cells were treated with CDTb (5.3 nM) in the absence of CDTa with or without increasing concentrations of α-defensin-5 (1, 3, 6 µM) for 4 h at 37°C. Representative images are shown in the left panel. After the incubation time, a MTS cell viability assay was performed. Values are given as mean ± SD (n=3). Significance was determined using the one-way ANOVA test (n.s. = not significant, ****p < 0.0001). **(D)** Caco-2 cells were seeded in an 8-well ibidi plate and pretreated with Fura-2AM (3 µM) for 45 min. Next, baseline was measured for 2 min, and the cells were then treated with CDTb (13 nM) in the presence or absence of α-defensin-5 (6 µM) as indicated.

Finally, the underlying molecular mode of inhibition was analyzed in more detail. For TcdA as well as for CDTa, no influence on the *in vitro*-enzymatic activity in the presence of α-defensin-5 could be observed (**Figure 4A**). Based on previous experiments, the capability of α-defensin-5 to precipitate TcdA was investigated. When incubated without inhibitor, TcdA was nearly completely present in the supernatant fraction. In the presence of α-defensin-5, the effect was inverted and TcdA was mainly detectable in the pellet fraction (**Figure 4B**). For CDT, the underlying mechanism might be different and is most likely based on the impact of α-defensin-5 on the pore-forming activity of CDTb or more precisely on the inactivation of the cytotoxic CDTb-pore. In higher concentrations, CDTb is able to induce pores in the plasma membrane in the absence of the enzymatic component CDTa. The pore-formation leads to dramatic changes in cell morphology and cell viability. Both effects could efficiently be prevented by the addition of α-defensin-5 (**Figure 4C**). These findings were confirmed using the Ca^2+^-imaging method in combination with living Caco-2 cells. Here, the increase in the Fura2 340/380 ratio, indicating the formation of CDTb-pores and the influx of Ca^2+^-ions into the cytosol of Caco-2 cells, could be prevented in the presence of α-defensin-5 (**Figure 4D**).

## Discussion

For CDAD, as well as for many other important diseases that are caused by exotoxins that are released from bacteria in the human body, the efficient targeted inhibition of these toxins besides the application of antimicrobial drugs is of highest relevance because the toxins cause the disease. The optimal inhibitors should affect the invading bacteria but also the released exotoxins. Such properties have been reported for human α-defensins. The α-defensins belong to the group of AMPs and are distributed in large amounts in host defense cells and tissues. In contrast to other alpha-defensins, such as α-defensins-1–4, which are predominantly produced in neutrophilic granulocytes and are therefore also called human neutrophil-derived α-defensins (HNP)1–4, the here investigated α-defensin-5 is mainly produced by enteric Paneth cells (Lehrer et al., 1993). Paneth cells are specialized cells at the base of intestinal crypts, also known as crypts of Lieberkühn. As part of the local immune system in the small intestine, Paneth cells release α-defensin-5 (and -6) into the lumen of the crypts preventing local excessive colonization of microbes (Selsted and Ouellette, 1995). Like all defensins, α-defensin-5 contains six intramolecular cysteine residues which form an unalterable and specific pattern of disulfide-bridges (Selsted and Harwig, 1989). The defined arrangement of the intramolecular disulfide-bridges is accountable for a conserved backbone topology, that protects the small peptide from proteolysis and maintains the function of α-defensin-5 as broad-spectrum microbicide in the environment of the intestinal lumen (Alpha Defensin - an overview | ScienceDirect Topics). Therefore, microbicidal tissue concentrations between 0.5 and 2.5 mg/g can be achieved in the mucosa of the ileum (Ghosh et al., 2002). It became evident that in addition to their microbicidal activity, specific human defensins are able to inactivate and neutralize several bacterial toxins (Kim et al., 2005; Kim et al., 2006; Giesemann et al., 2008; Lehrer et al., 2009). Based on these previous findings, the protective role of α-defensin-5 against TcdA, TcdB, and CDT was investigated in this present study.

For TcdA, TcdB, and CDT, a time- and concentration-dependent inhibition by α-defensin-5 was observed. For all three toxins, the inhibition by α-defensin-5 could be quantified morphologically on Vero cells and biochemically on Caco-2 cells. By using fluorescence microscopy, the inhibitory potency of α-defensin-5 could be further strengthened. This fact could also be substantiated for the medically relevant combination of all three toxins together. However, the underlying inhibition mechanism seems to differ between the large clostridial glucosylating toxins TcdA and TcdB and the binary actin ADP-ribosylating toxin CDT. In the presence of the inhibitor we observed a clear precipitation of TcdA, which could be made visible in a sodium dodecyl sulfate polyacrylamide gel. At the same time, we could not detect any influence of α-defensin-5 on the enzymatic activity of TcdA, which is consistent with our earlier results for α-defensin-1 (Fischer et al., 2020) but in some contrast to the work of other groups. Giesemann et al. showed in an earlier study that both α-defensin-1 and α-defensin-5 can effectively inhibit TcdB, but not TcdA. In our present study, however, we clearly show inhibition of TcdA in the presence of α-defensin-5. In direct comparison, a broader concentration range of α-defensin-5 was tested in our study, and in addition, a five-fold less concentration of TcdA and recombinant instead of native TcdA was used. Giesemann et al. could show that at least for α-defensin-1, the inhibition of TcdB was mediated by negatively influencing the glucosyltransferase activity of TcdB whereas for α-defensin-5, less or almost no influence on the glycosyltransferase activity of the toxin was observed. This is in line with our findings of the present study, where also no influence of α-defensin-5 on the enzyme activity of TcdA was detected. Au contraire, we found, that binding and co-precipitation of TcdA is the underlying inhibitory mechanism of α-defensin-5. Extensive co-precipitation of TcdB with α-defensin-5 was also observed in the work of Giesemann et al. and in the present study we could show, that this is also the underlying mode of inhibition for TcdA. Presumably, native TcdA that was used by Giesemann et al. was more extensively bound by protein impurities that might have prevented successful interaction with α-defensin-5 (Giesemann et al., 2008).

For CDT, the mode of inhibition seems to be based on the inactivation of the CDTb-pore. Several binding/transport components of binary bacterial toxins form cation-selective channels to transport the enzymatically active subunits of such toxins into cells (Schmid et al., 1994). It was demonstrated that also CDTb forms pores in lipid bilayer membranes *in vitro* (Kronhardt et al., 2017). More recently, it was shown that CDTb forms di-heptamer like structures (Xu et al., 2020). Furthermore, when applied to cells, CDTb alone (in the absence of CDTa) is able to cause dramatic changes in cell morphology and cell viability (Kronhardt et al., 2017). These massive cell-damaging effects could be completely prevented by the addition of α-defensin-5. But also for CDT, we did not find any influence of the small peptide on the enzymatic activity, although this has already been described for other toxins of the mono-ADP-ribosyltransferase family (Kim et al., 2006). Most likely, α-defensin-5 is not able to block existing CDTb pores like other known pore blockers such as chloroquine (Schmid et al., 1994) or other related compounds like fluphenazine (Bachmeyer et al., 2001; Bachmeyer et al., 2003). Based on our previous finding (Fischer et al., 2020), we assume the same underlying mode of inhibition for α-defensin-5 as for α-defensin-1, namely the prevention of the formation of new CDTb-pores by α-defensin-5. This conclusion could be drawn on the basis of analyzing cell morphology and viability as well as on the basis of calcium imaging. In these experiments, a clear increase in intracellular Ca^2+^ was observable when Caco-2 cells were treated with CDTb in calcium containing medium. This effect was prevented in the presence of α-defensin-5 indicating the successful prevention of the formation of CDTb-pores. Interestingly, when Caco-2 cells were treated with CDTb in calcium-free medium, no increase in intracellular Ca^2+^ could be detected, suggesting that CDTb does not lead to the release of intracellular calcium per se (data not shown).

Taken together, α-defensin-5 is a specific inhibitor of *C. difficile* toxins TcdA, TcdB, and CDT. This human peptide might be an auspicious pharmacological inhibitor to treat/prevent CDAD, in particular after infection with hypervirulent, CDT-producing strains of *C. difficile*. Thereby, a beneficial role of α-defensin-5 might be a first line host defense mechanism against invading pathogens in combination with a newly discovered inhibitory potency against the produced protein toxins.

## Data Availability Statement

The raw data supporting the conclusions of this article will be made available by the authors, without undue reservation, to any qualified researcher.

## Author Contributions

SF designed, supervised, and analyzed experiments and wrote the manuscript. MK and ML performed experiments. PP and KA provided toxins, analyzed data, and proof-read the manuscript. HB wrote the manuscript and supervised the study.

## Funding

The work in the Barth group was financially supported by the Deutsche Forschungsgemeinschaft project number 316249678- SFB 1279 (project C02). ML is a fellow of the Promotionsprogramm Experimentelle Medizin of the International Graduate School in Molecular Medicine in Ulm.

## Conflict of Interest

The authors declare that the research was conducted in the absence of any commercial or financial relationships that could be construed as a potential conflict of interest.
